# Supplementary figures and images for: SuperMetal: a generative AI framework for rapid and precise metal ion location prediction in proteins
Source: J Cheminform. 2025 Jul 15;17:107. doi: 10.1186/s13321-025-01038-9 (PMC12265342; doi:10.1186/s13321-025-01038-9)

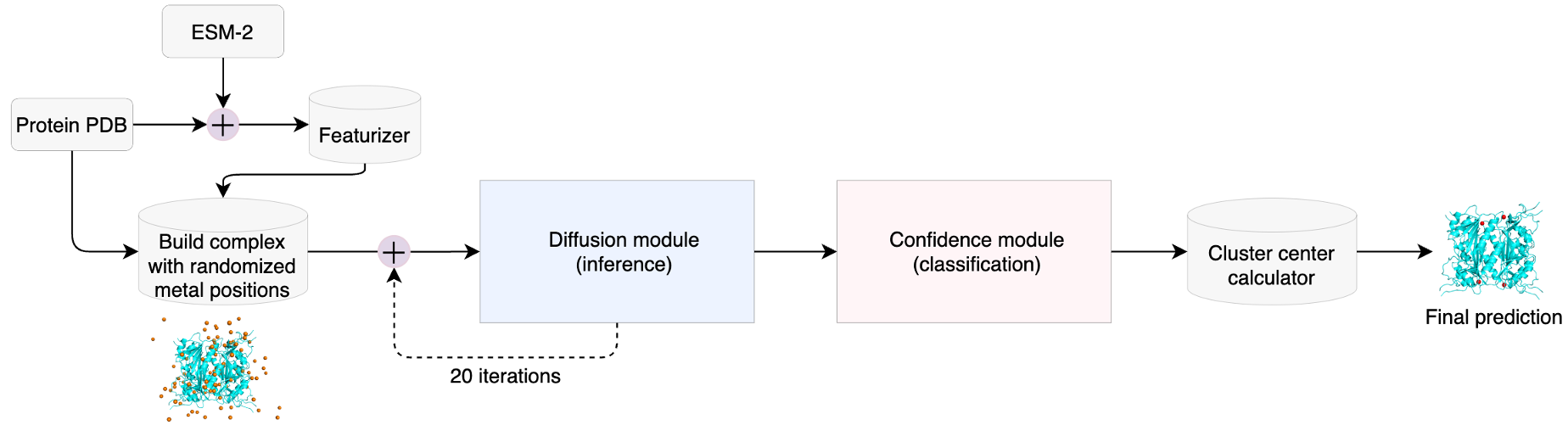

Supplement: Supplementary file 1 — Supplementary material 1 [file 13321_2025_1038_MOESM1_ESM.zip › SI_0211/figures/general_inference_workflow.png]

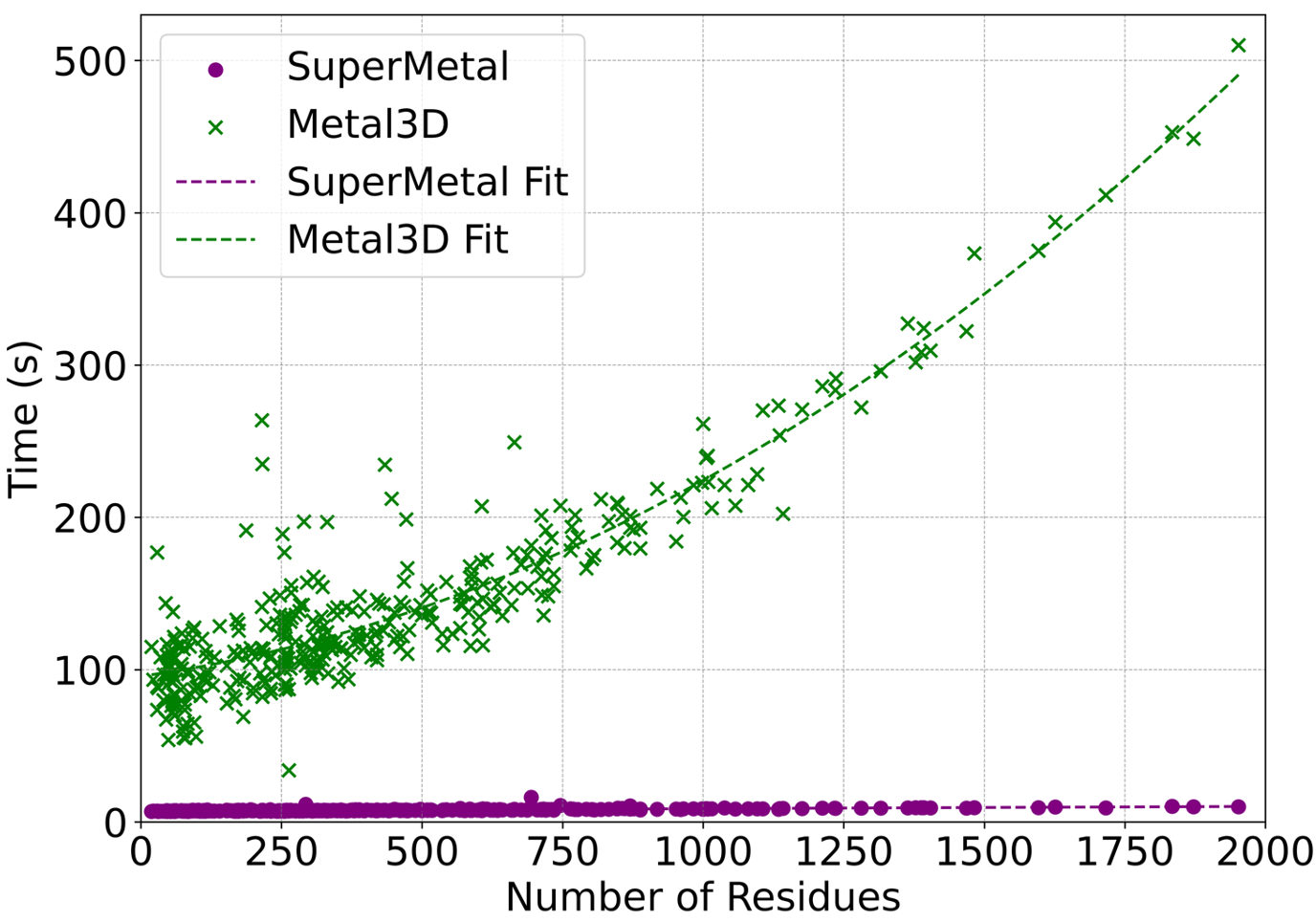

Supplement: Supplementary file 1 — Supplementary material 1 [file 13321_2025_1038_MOESM1_ESM.zip › SI_0211/figures/speed.png]

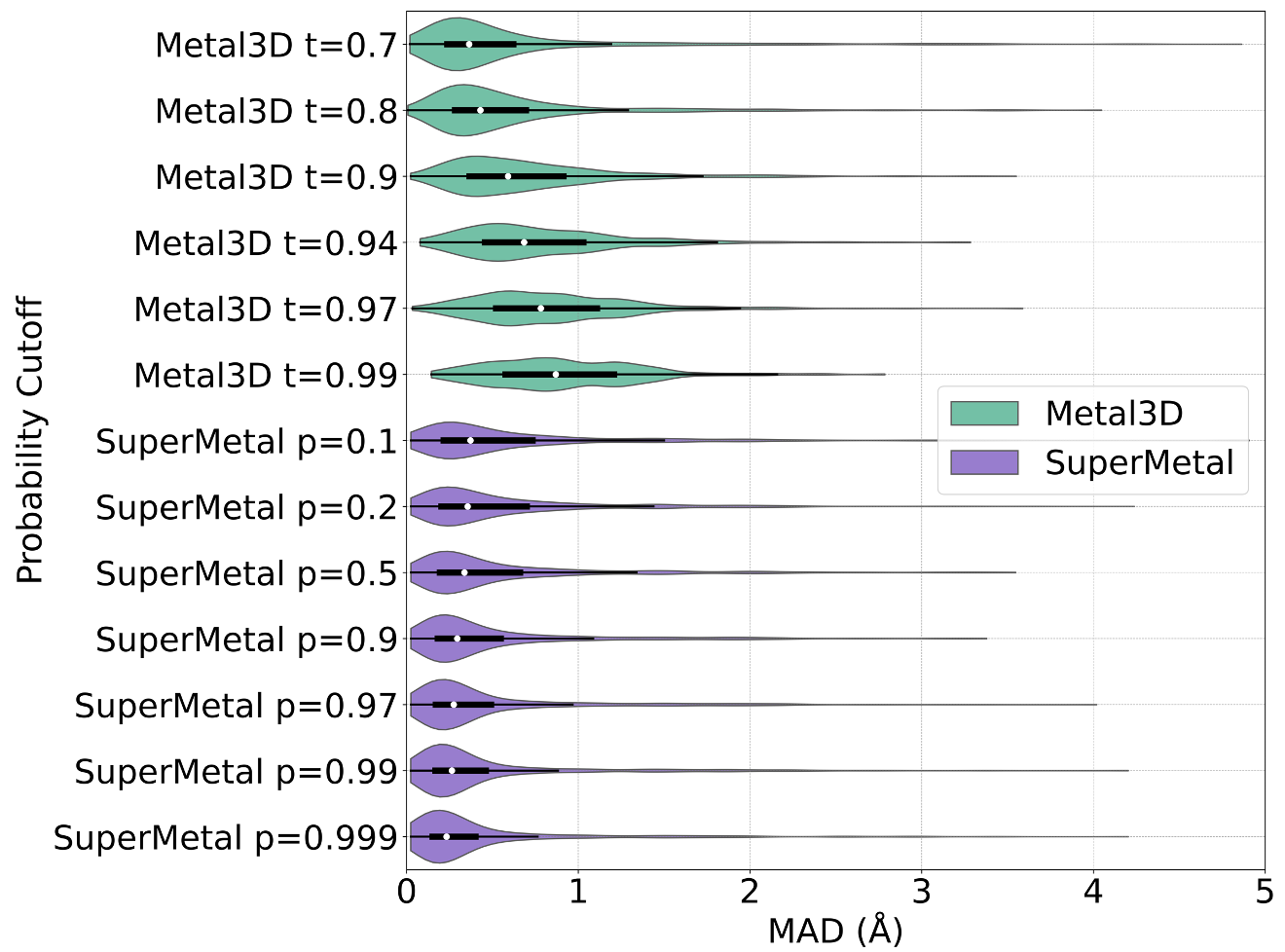

Supplement: Supplementary file 1 — Supplementary material 1 [file 13321_2025_1038_MOESM1_ESM.zip › SI_0211/figures/mad.png]

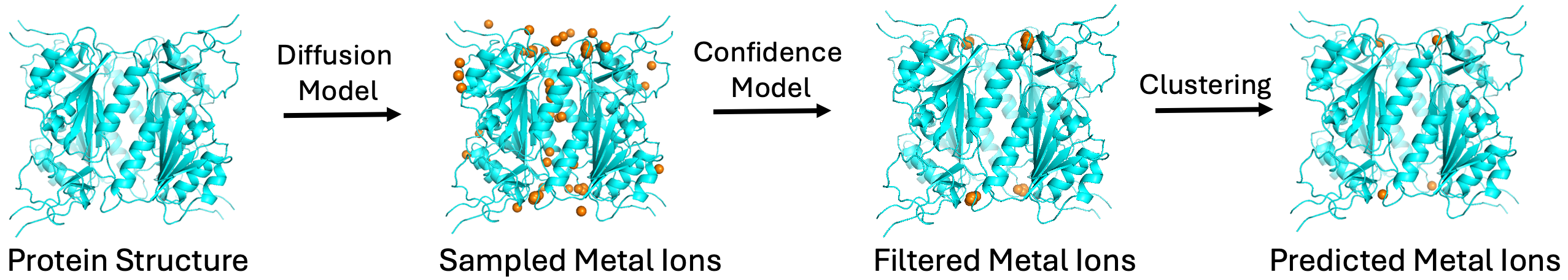

Supplement: Supplementary file 1 — Supplementary material 1 [file 13321_2025_1038_MOESM1_ESM.zip › SI_0211/figures/fig_overview.png]

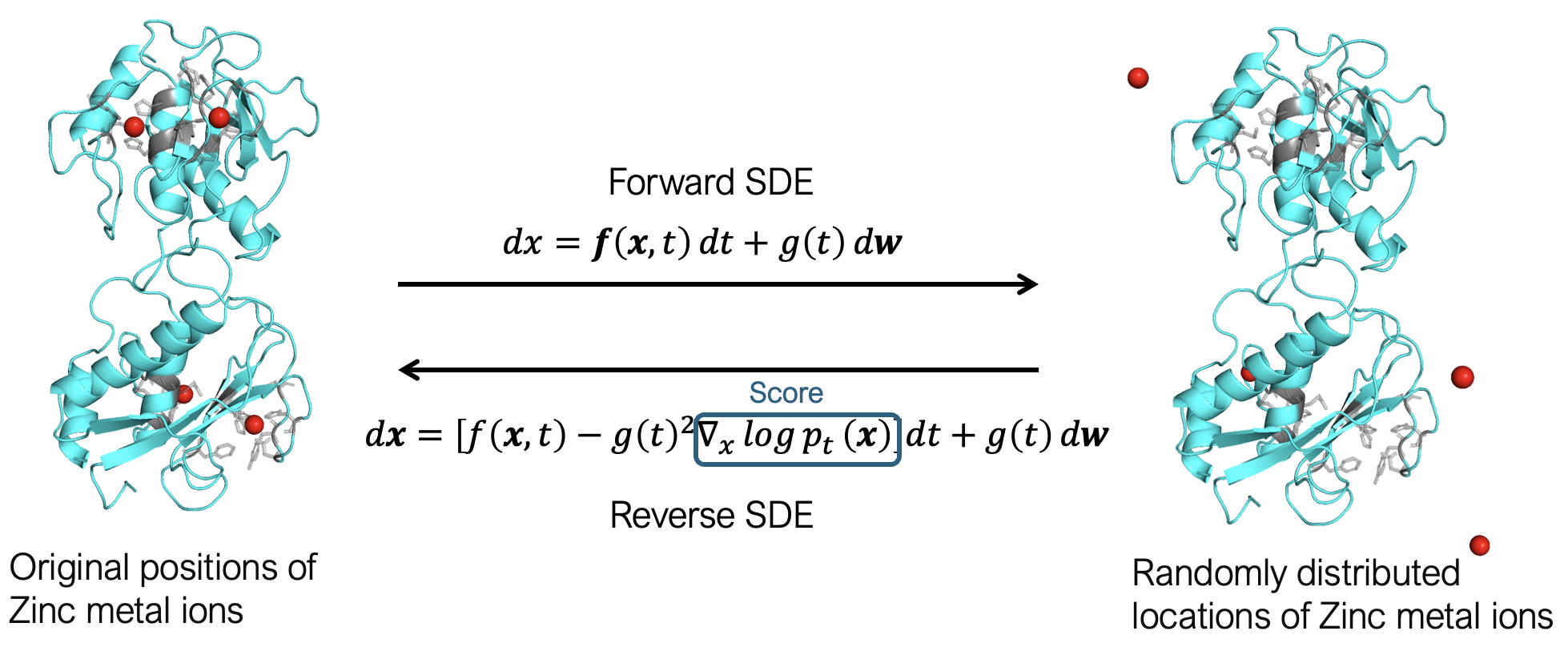

Supplement: Supplementary file 1 — Supplementary material 1 [file 13321_2025_1038_MOESM1_ESM.zip › SI_0211/figures/diffusion_theory.png]

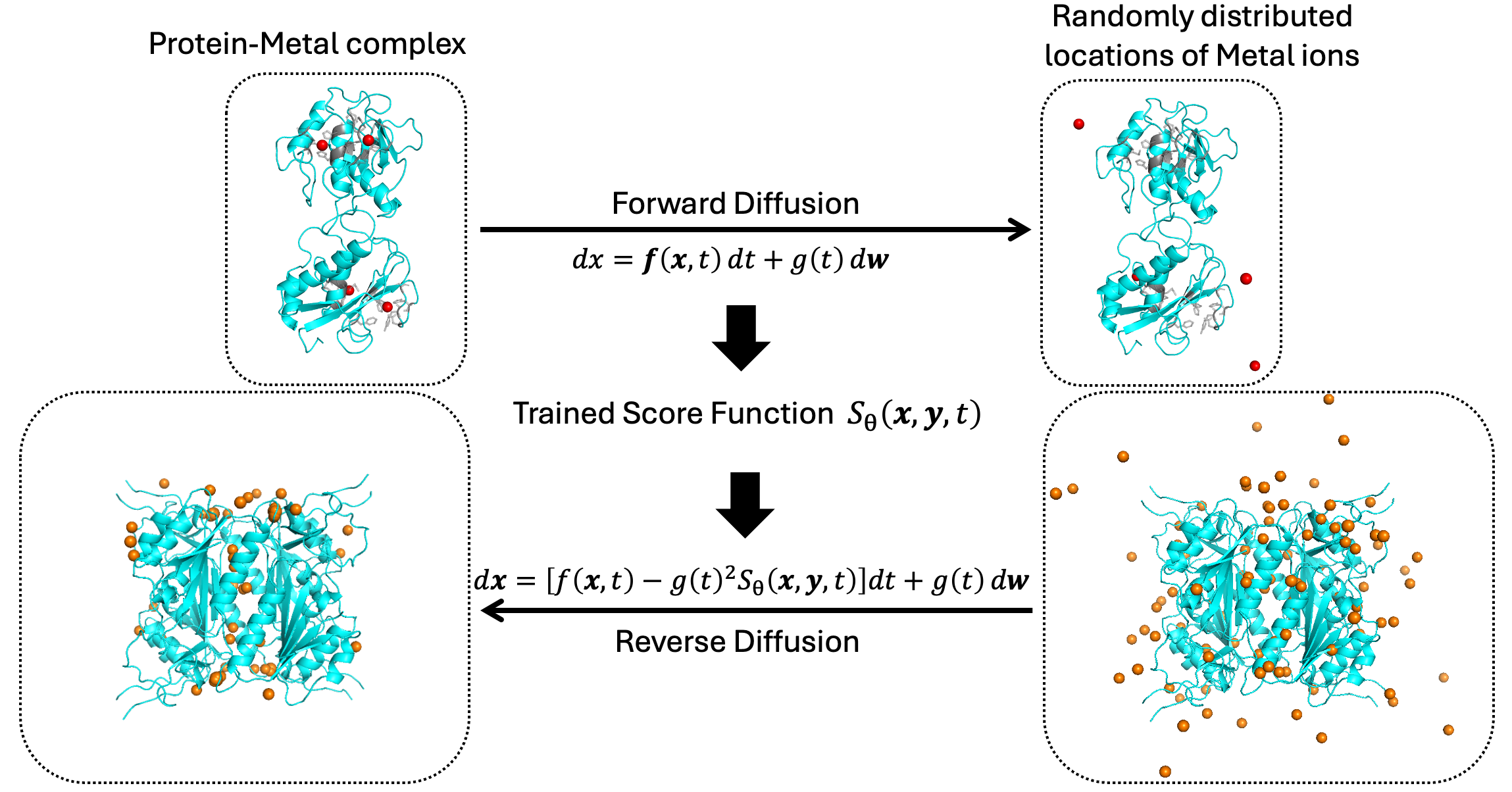

Supplement: Supplementary file 1 — Supplementary material 1 [file 13321_2025_1038_MOESM1_ESM.zip › SI_0211/figures/diffusion_theory_supermetal.png]

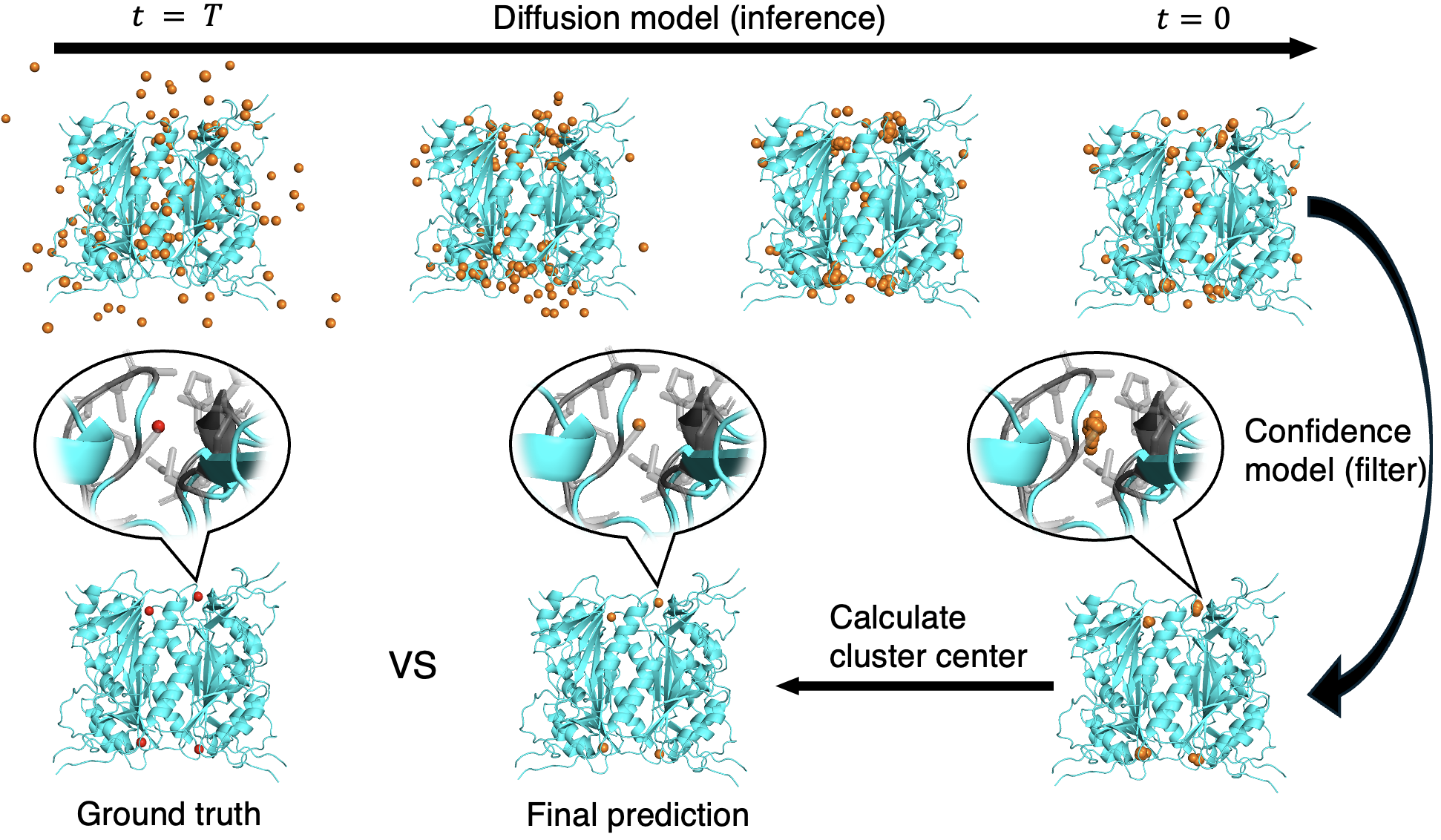

Supplement: Supplementary file 1 — Supplementary material 1 [file 13321_2025_1038_MOESM1_ESM.zip › SI_0211/figures/visualization_workflow.png]

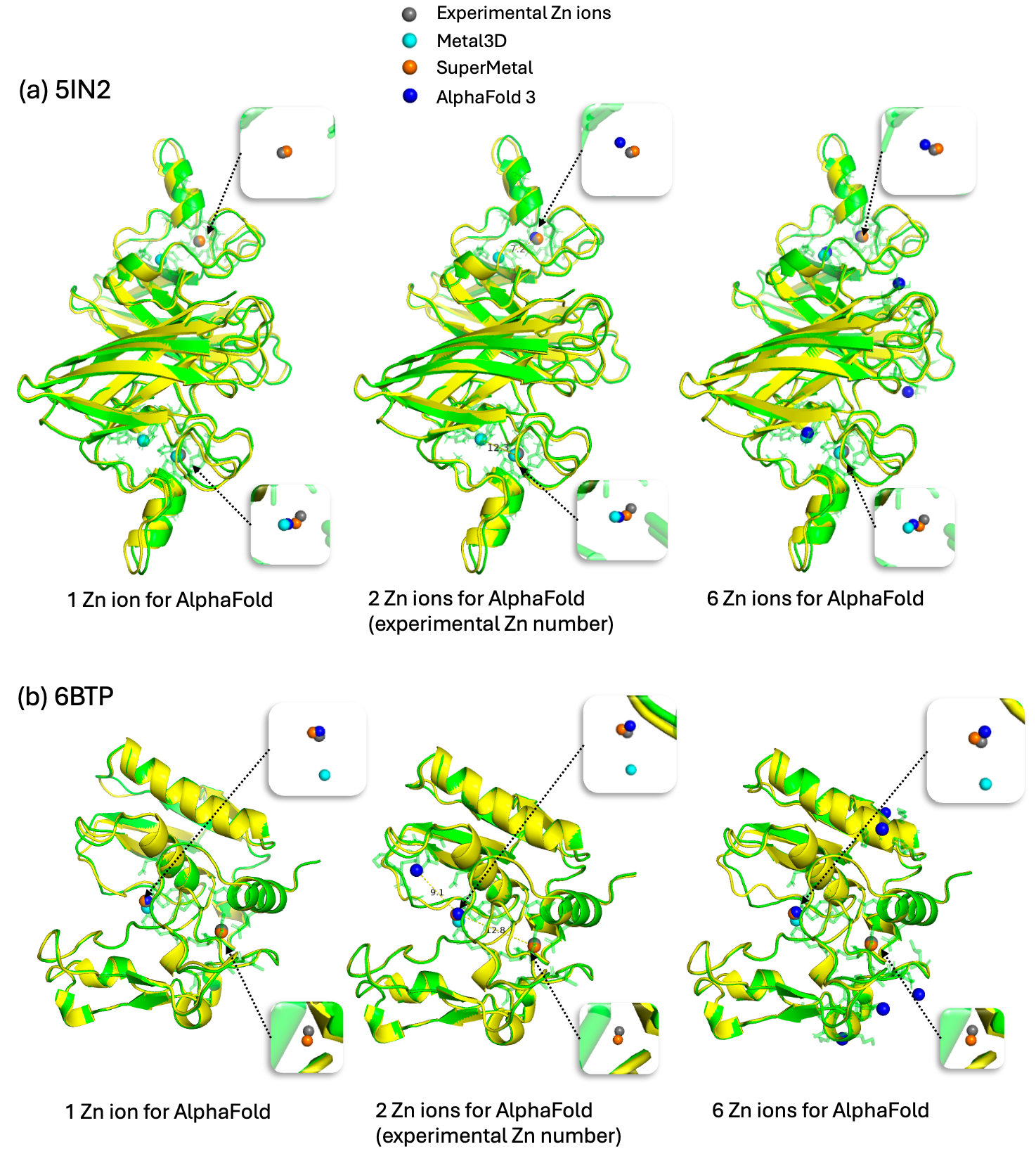

Supplement: Supplementary file 1 — Supplementary material 1 [file 13321_2025_1038_MOESM1_ESM.zip › SI_0211/figures/case_study.png]

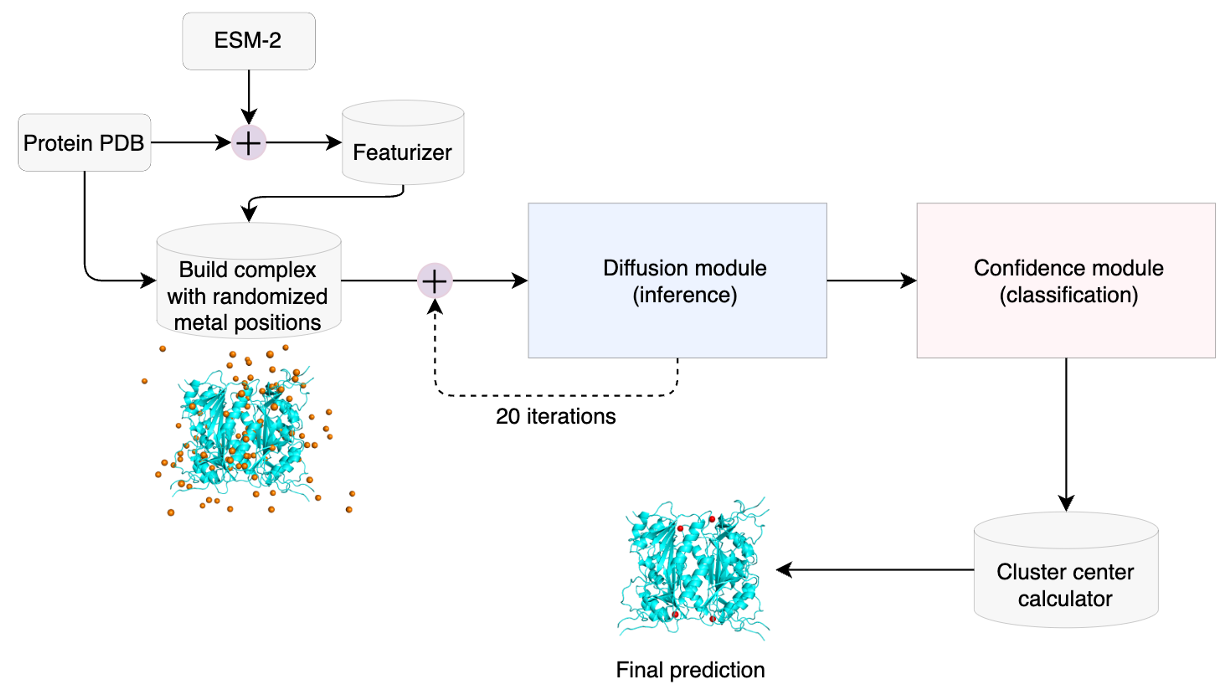

Supplement: Supplementary file 1 — Supplementary material 1 [file 13321_2025_1038_MOESM1_ESM.zip › SI_0211/figures/general_flow.png]

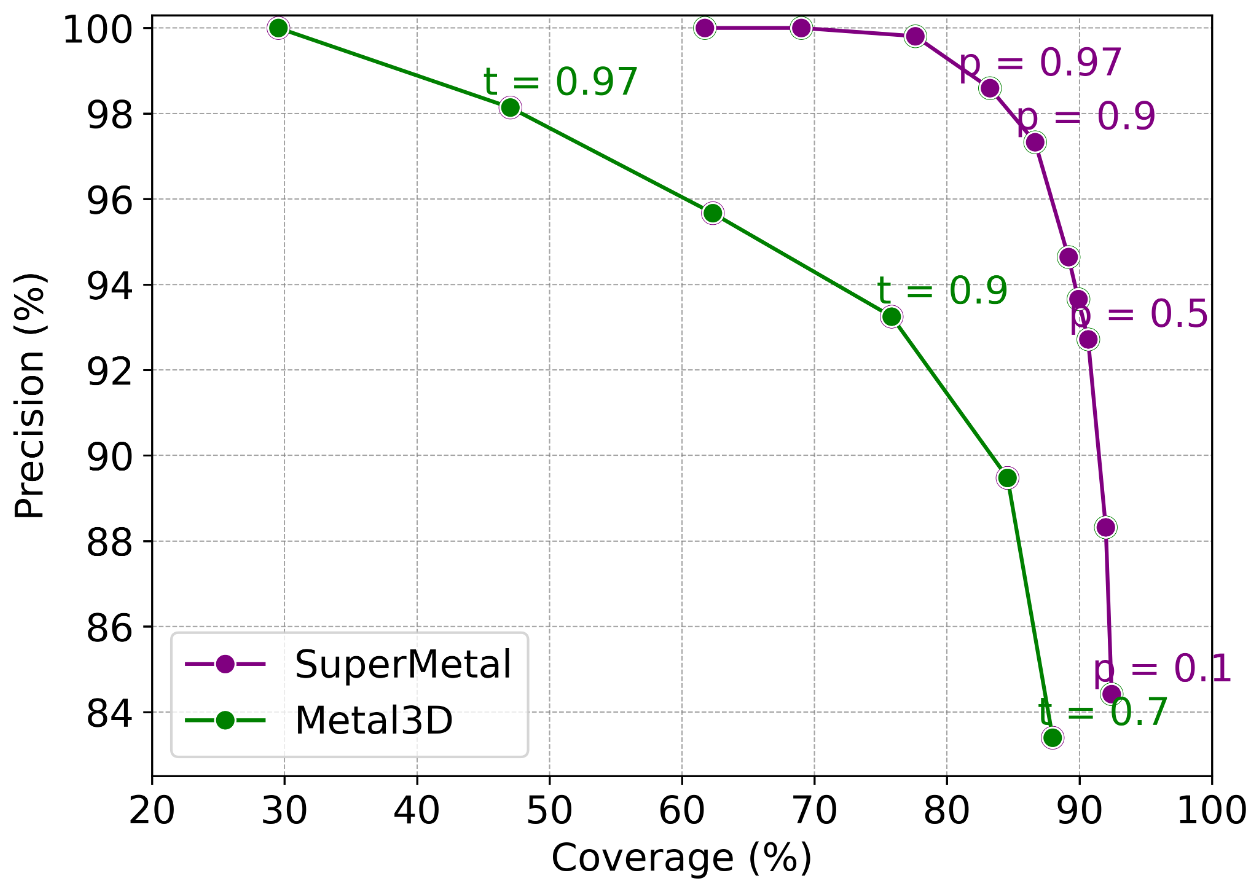

Supplement: Supplementary file 1 — Supplementary material 1 [file 13321_2025_1038_MOESM1_ESM.zip › SI_0211/figures/pr.png]

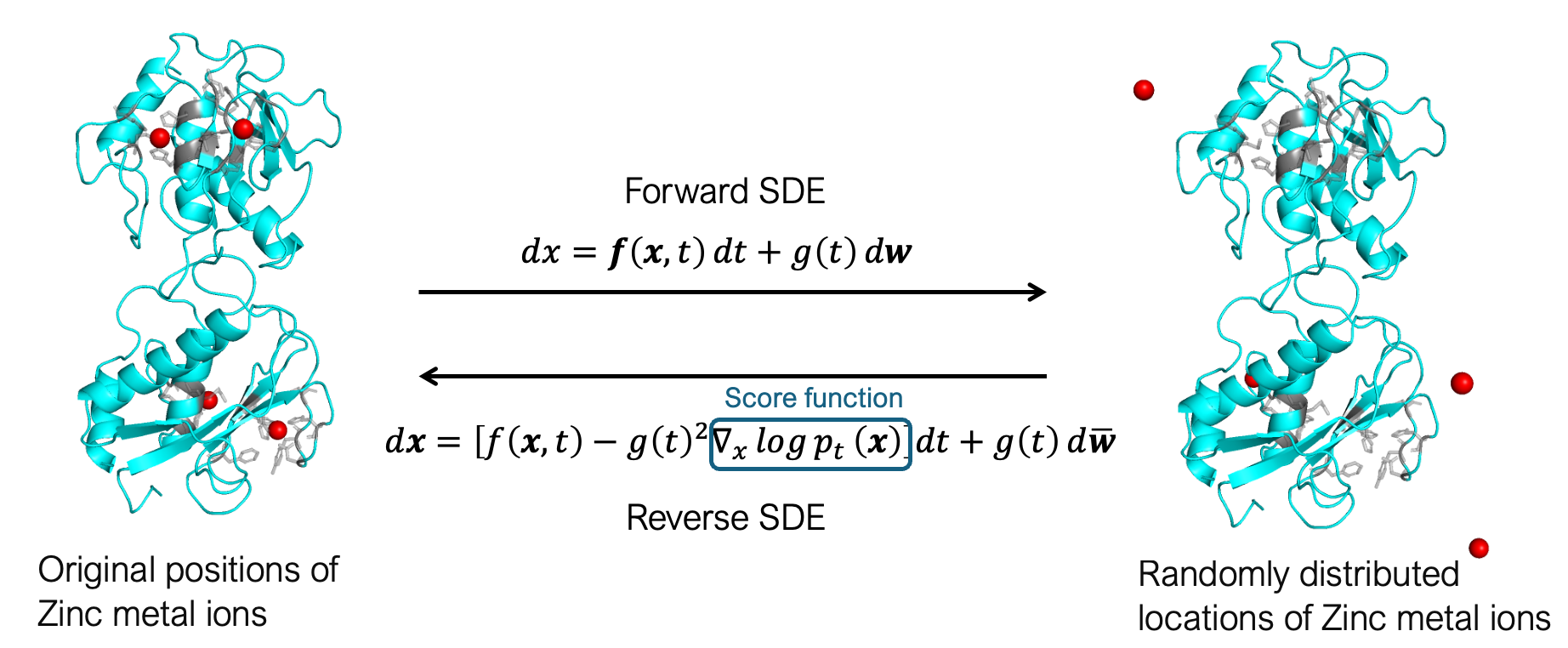

Supplement: Supplementary file 1 — Supplementary material 1 [file 13321_2025_1038_MOESM1_ESM.zip › SI_0211/figures/diffusion_theory_backup.png]

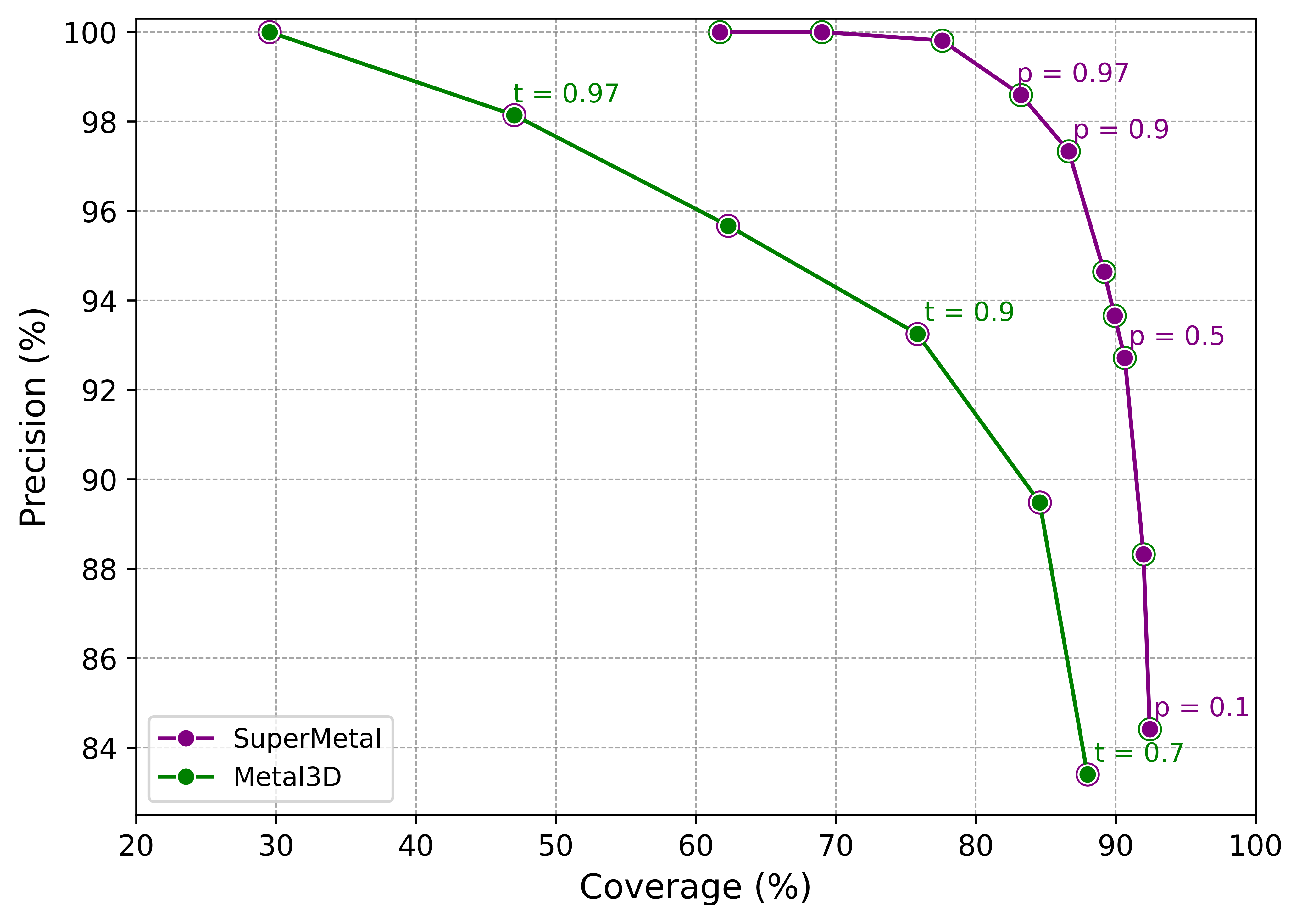

Supplement: Supplementary file 1 — Supplementary material 1 [file 13321_2025_1038_MOESM1_ESM.zip › SI_0211/figures/fiigure_pr.png]

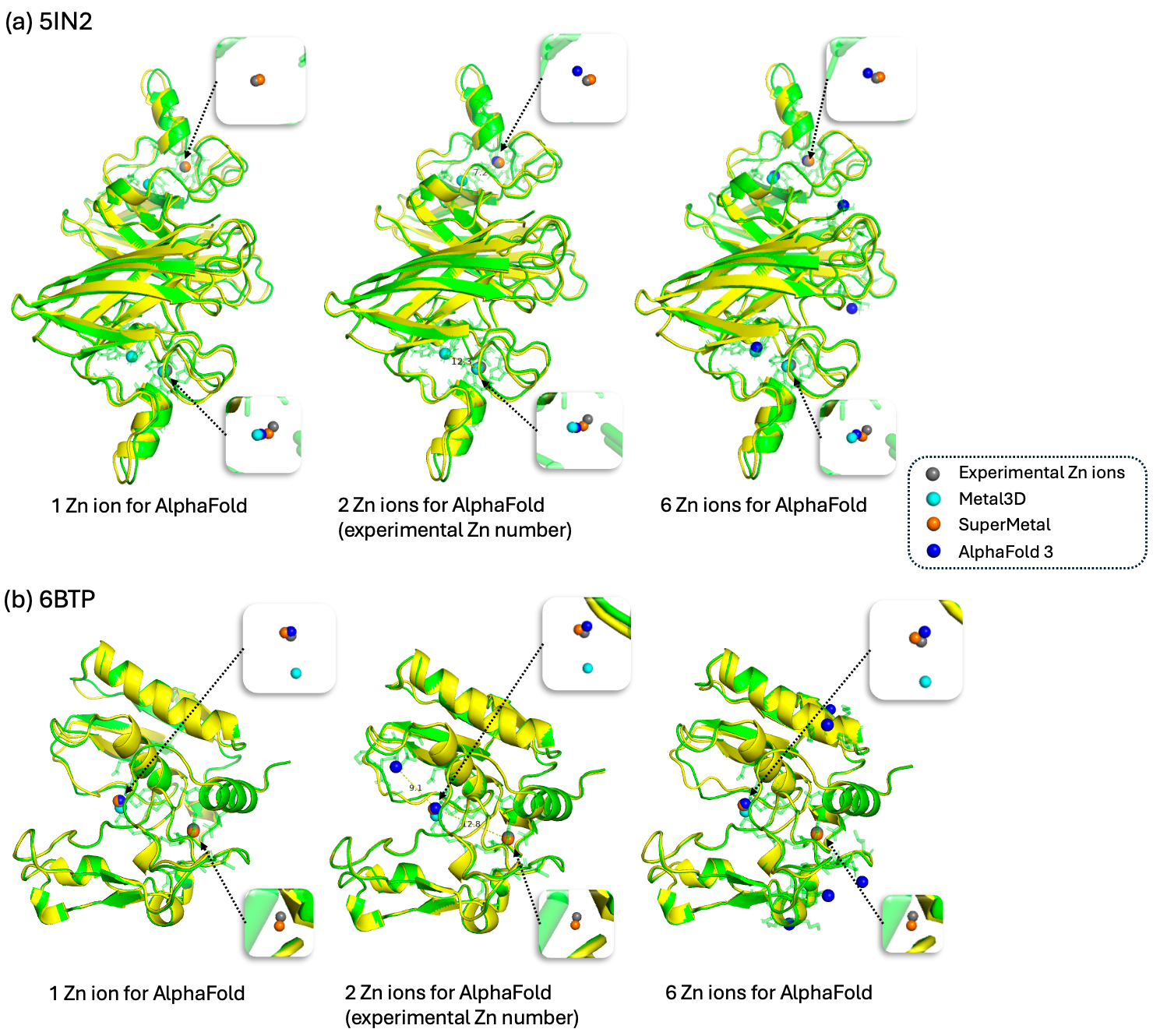

Supplement: Supplementary file 1 — Supplementary material 1 [file 13321_2025_1038_MOESM1_ESM.zip › SI_0211/figures/case_study_2.png]

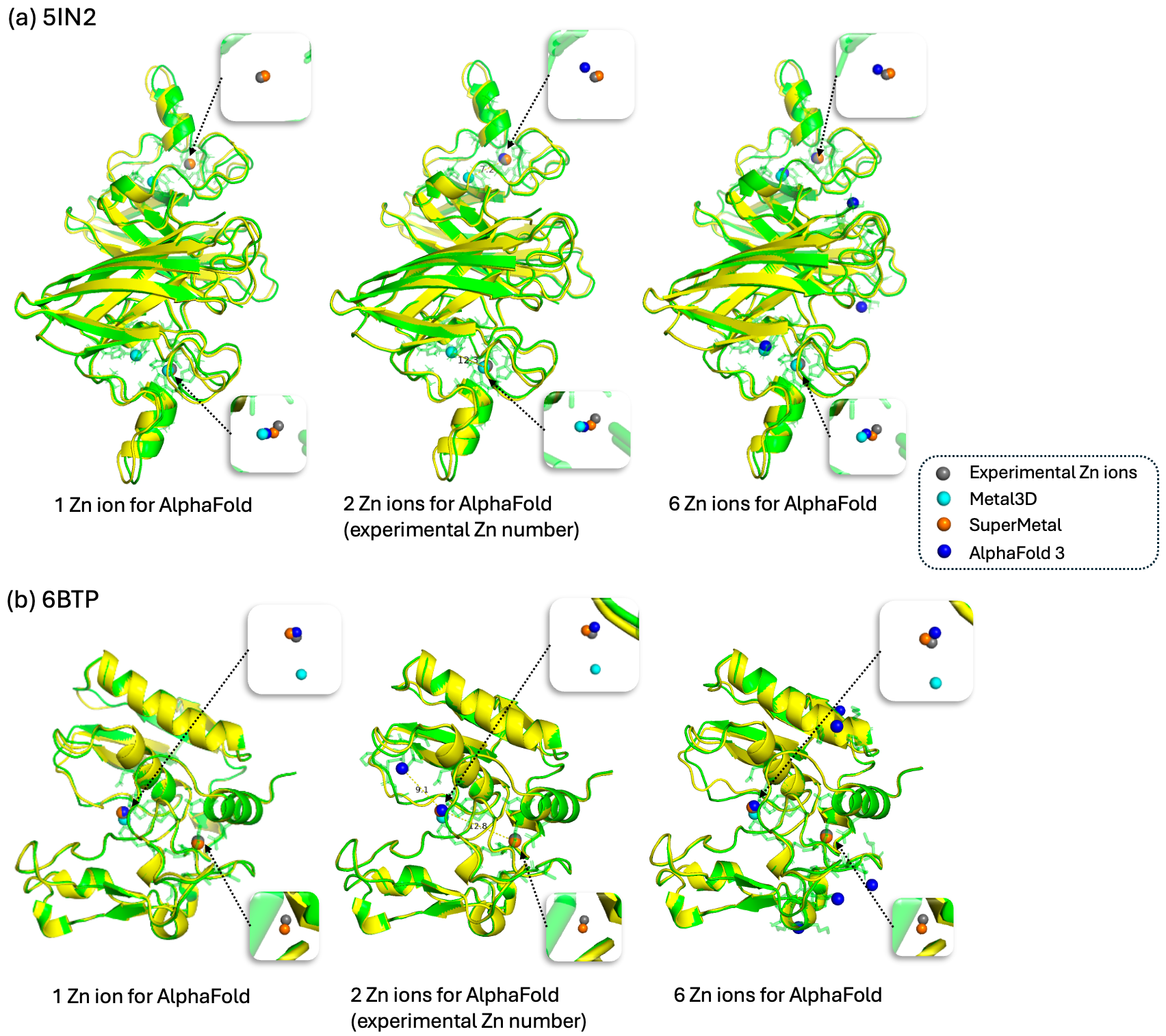

Supplement: Supplementary file 1 — Supplementary material 1 [file 13321_2025_1038_MOESM1_ESM.zip › SI_0211/figures/case_study_3.png]

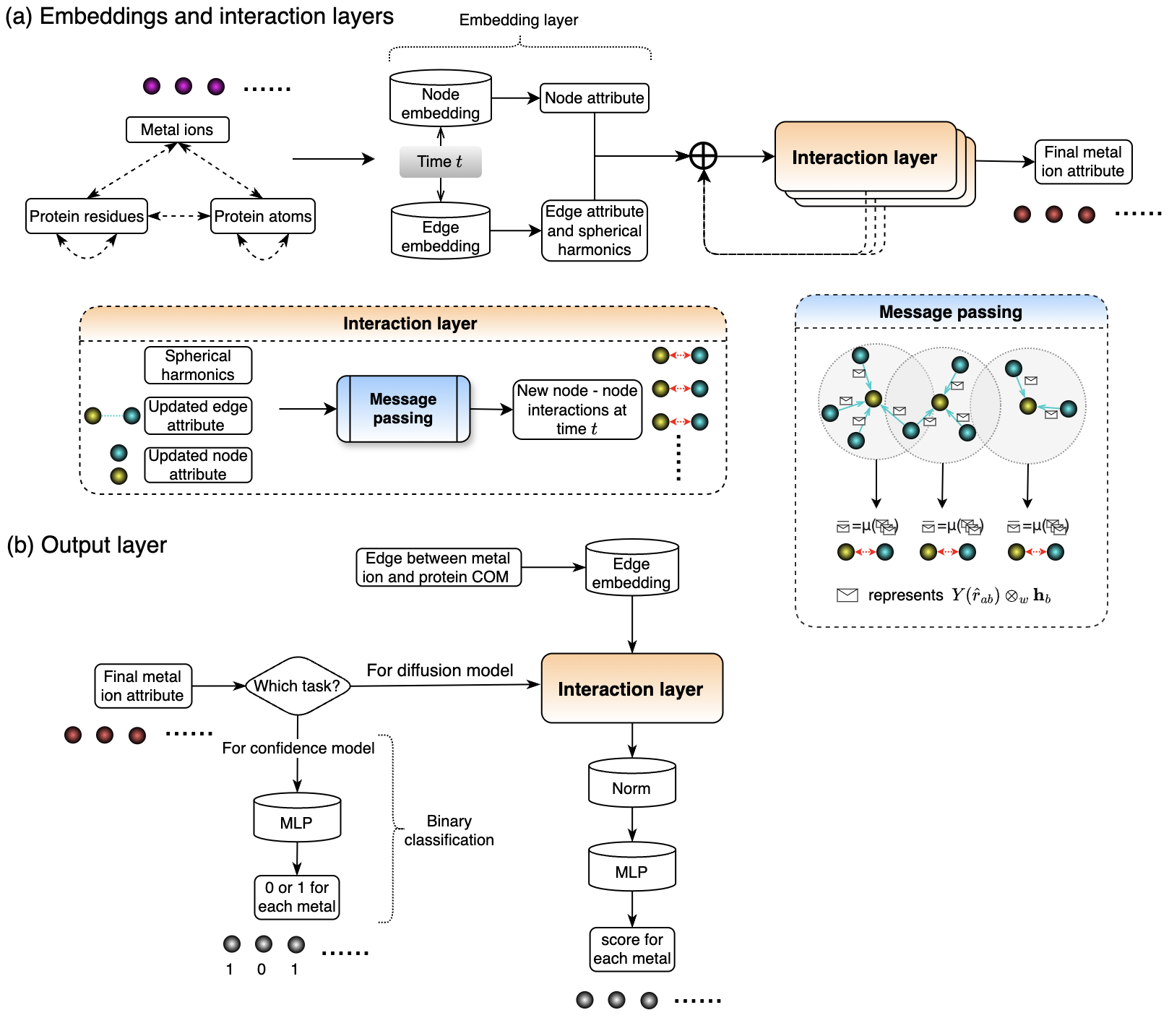

Supplement: Supplementary file 1 — Supplementary material 1 [file 13321_2025_1038_MOESM1_ESM.zip › SI_0211/figures/architecture.png]
